# Regional-scale biogeographical patterns of soil- and root-associated microbial communities across nine planted Chinese fir forests

**DOI:** 10.1128/msphere.00450-25

**Published:** 2025-07-31

**Authors:** Feihua Zhou, Hanshuo Zhang, Wen Zhong, Hao Yang, Luhong Zhou, Zhi-Jie Yang, Yalin Hu, Yong Zheng

**Affiliations:** 1 Key Laboratory for Humid Subtropical Eco-geographical Processes of the Ministry of Education, Fujian Normal University 12425, Fuzhou, China; 2 Shanghang Mountain Forest Carbon Sink Observation and Research Station of Fujian Province, Fujian Normal University, Longyan, China; 3 Fujian Sanming Forest Ecosystem National Observation and Research Station, Fujian Normal University, Sanming, China; 4 Forest Ecology Stable Isotope Center, College of JunCao Science and Ecology, Fujian Agriculture and Forestry University 12449, Fuzhou, Fujian, China; University of Wisconsin-Madison, Madison, Wisconsin, USA

**Keywords:** biomass, Chinese fir plantation, community composition, compartment, diversity, microbiome

## Abstract

Chinese fir (*Cunninghamia lanceolata*) is an economically important plantation tree species. Gaining insights into the belowground microbiome of Chinese fir is critical for optimizing plantation management and enhancing timber production. In this study, we investigated microbial community structures in both rhizospheric soil and root samples from nine Chinese fir plantations (sites) at a regional scale. Moreover, we analyzed relationships between tree growth and microbial community structures and soil properties. Our results revealed that significantly higher bacterial and fungal richness was observed in rhizospheric soils than in tree roots. Differing distribution patterns of soil- and root-associated bacterial and fungal community compositions were observed across different sites. Soil nitrate was the key factor shaping root-associated bacterial diversity, and both soil total nitrogen and nitrate were the critical drivers influencing root-associated fungal community composition. There were apparent geographical variations in the biomass and growth increment of Chinese fir trees, with soil moisture emerging as the strongest predictor for these two parameters. Moreover, soil-associated bacterial community composition, root-associated bacterial diversity, and root-associated fungal community composition were identified as the primary determinants of tree biomass. Our findings highlight the critical but different contributions of soil- and root-associated bacterial and fungal communities to the productivity of trees in subtropical plantations.

**IMPORTANCE:**

Chinese fir plantations are widely distributed in Southeast China and characterized by their considerable economic significance. Belowground microbial communities play pivotal roles in shaping forest ecosystem functions. Nevertheless, knowledge of the relationship between microbial communities and tree growth is scarce. Here, we investigated soil- and root-associated bacterial and fungal communities and their relationships with the tree growth of nine Chinese fir plantations in subtropical regions. We found that both compartment and site factors influenced bacterial and fungal diversity and community composition. Apparent geographical variations in the biomass and growth increment of Chinese fir trees were observed. Moreover, soil-associated bacterial community composition, root-associated bacterial diversity, and fungal community composition were identified as the primary determinants of tree biomass. Altogether, this study provides a comprehensive analysis of microbial communities in mature Chinese fir planted forests, offering new insights into their roles in supporting forest productivity.

## INTRODUCTION

Forests play a pivotal role in mitigating global warming and maintaining global climate stability, primarily by balancing the global carbon budget (1, 2). Forest carbon storage accounts for 82.5% of total terrestrial vegetation carbon storage, making it a crucial indicator of the carbon sequestration potential of forests (3). Consequently, afforestation programs have become an essential tool for enhancing ecological carbon sequestration, leading to a global increase in plantation areas (4). Afforestation leads to changes in soil properties, litter quantity and quality, as well as root exudates, which, in turn, exert substantial effects on the balance between the carbon content in vegetation and soil (5, 6). These changes result in profound effects on soil- and root-associated microbial communities (7). The intricate interplay between microbial composition, enzyme activities, and carbon use efficiency is strongly intertwined with soil carbon accumulation, playing a pivotal role in the overall health and productivity of forest ecosystems (8, 9).

Soil microorganisms are indispensable in biogeochemical cycling processes, such as carbon decomposition, nitrogen cycling, and phosphorus mineralization (10, 11). Global warming is expected to accelerate the microbial decomposition of soil carbon (12–14), and the loss of microbial diversity constrains the capacity of soils to mitigate climate change (15). Given their significance, soil microbial diversity is therefore regarded as a bioindicator of ecosystem functions (16). It offers a means for assessing the status of tree growth, overall health of forests, and effectiveness of climate change mitigation. Moreover, trees interact with an enormous diversity of microbes within their roots and rhizospheric soil. These complex tree-associated bacterial and fungal communities are crucial for tree health, forest productivity, and overall ecosystem functioning (17–19). For example, root-associated bacteria or fungi can enhance disease resistance, possibly by suppressing soil-borne pathogens or stimulating the plant immune system (20, 21), resulting in increased growth and carbon sequestration. To improve the predictability of forest carbon storage, it is imperative to elucidate the relationship between rhizospheric microbial communities and tree biomass.

The significant role of rhizospheric microorganisms in facilitating tree growth has been the focus of numerous previous studies. For example, it has been reported that phylogenetically diverse arbuscular mycorrhizal (AM) fungi significantly enhance the growth of seedlings of native potential crop trees (22) and that ectomycorrhizal fungal composition is a strong bio-indicator of the underlying drivers of tree growth. Moreover, it has also been reported that variations in the abundance and structure of ectomycorrhizal fungal communities cause differences in tree growth (23). Additionally, Yu et al. (24) further observed distinct relationships between various fungal species and the annual growth rate and wood density of white spruce trees. Similarly, Dash et al. (25) found that the biomass of *Dalbergia sissoo* Roxb. seedlings was higher under inoculated conditions than under control conditions. However, most previous studies have primarily focused on the seedlings of various tree species using potting experiments (22, 25). In contrast, research on mature and typical tree species in subtropical regions remains limited. Therefore, it is imperative to conduct comprehensive research to uncover the intricate mechanisms through which root- and soil-associated microbiomes impact the growth and biomass of specific trees in subtropical areas.

Chinese fir, *Cunninghamia lanceolata*, is a fast-growing, evergreen coniferous tree species that has been widely planted in Southeast China for over a millennium (26). Chinese fir plantations are widely developed and account for 11.9% of the total plantation area in China because of their considerable economic significance (27, 28). However, the extensive and long-term planting of Chinese fir results in soil acidification and decreased fertility (29, 30), which consequently decreases tree productivity and carbon stocks. Therefore, for the improved sustenance of the productivity and carbon sequestration potential of forests, it is highly necessary to reveal the critical factors impacting tree growth (31).

Despite the crucial role of microbial communities in sustaining soil fertility and carbon turnover, the impact of soil- and root-associated bacterial and fungal community structures on the aboveground biomass of Chinese fir trees remains poorly documented. Therefore, in this study, we aimed to explore the effects of biotic (microbes) and abiotic (soil properties and climate) factors on tree biomass. Based on the pivotal roles of microbial communities in maintaining tree health, productivity, and overall ecosystem functioning (17–19), coupled with the understanding that the structures of these communities are impacted by plant compartment and habitat conditions (32–34), we hypothesized that (i) microbial communities in different plant compartments would vary according to site and environmental factors, and (ii) these variations in microbial diversity would correlate with tree biomass and growth patterns. We sought to gain deeper insights into the roles of soil- and root-associated bacteria and fungi in impacting tree growth by examining these interactions at a regional scale, ultimately contributing to the sustainable management and biodiversity conservation of subtropical plantation ecosystems. Taken together, this study provides the first comprehensive analysis of microbial communities in mature Chinese fir plantations across nine sites in Southeast China, offering new insights into their roles in supporting forest productivity and carbon storage.

## MATERIALS AND METHODS

### Study site, experimental design, vegetation survey, and sample collection

The study site is located in Fujian Province, Southeast China (23°31′–28°18′ N, 115°50′–120°43′ E). Characterized by hilly and mountainous terrain, the region experiences a subtropical marine monsoon climate. For sampling purposes, representative Chinese fir plantations were selected across nine state-owned forest farms, including Baisha (BS), Guanzhuang (GZ), Jinshan (JS), Qiujiashan (QJS), Sanming (SM), Wuyi (WY), Wuyishan (WYS), Xiapu (XP), and Xiayang (XY). These sites were chosen based on their geographical spread across Fujian Province and uniform tree ages. All are key forest farms that contribute considerably to Fujian’s forestry industry. These forest farms have an average annual temperature ranging from 15.7°C to 20.1°C, accompanied by annual precipitation between 1,378 and 1,918 mm. Elevations vary from 203 to 729 m. The tested soil type is a typical granite acidic red soil, highly developed Oxisol derived from sandstone, which exhibits relatively low levels of available phosphorus (AP). The fundamental details of these nine forest farms are summarized in previous studies (35, 36).

The bulk soil, rhizospheric soil, and root samples were collected from the nine sites, i.e., forest farms, in August 2022. At each site, four distinct subsites (30 m × 30 m plots) were chosen. The age of the Chinese fir trees at these subsites ranges between 20 and 30 years. These subsites were spaced at least 200 m apart to ensure their spatial independence. Within each subsite, nine uniformly growing representative Chinese fir trees (spaced approximately 10 m apart) were randomly selected. Using a soil drill, four soil cores (15 cm deep, ø = 5 cm) were collected at 0.5 m intervals around the tree trunk in four directions. Twelve soil cores from three trees were pooled to form a composite sample, resulting in a total of three bulk soil samples per subsite. Overall, 12 bulk soil samples (3 × 4 plots) were collected from each site, leading to a total of 108 bulk soil samples (12 × 9 sites) collected from all nine sites. Additionally, rhizospheric soil and root samples (two sample types) were collected from the nine selected Chinese fir trees. Briefly, root samples were collected from every three tree individuals by tracing their roots from the base of the tree trunk, whereas rhizospheric soil samples were simultaneously obtained during root sampling. In total, 216 composite rhizospheric soil and root samples (two types × 9 sites × 4 plots × 3 samples) were obtained. All samples were transported to the laboratory in an ice box for further processing. Fresh soil samples were sieved through a 2 mm sieve to remove stones, visible roots, and debris and then stored at 4°C until physicochemical analysis. Soil and root samples were preserved at −80°C until DNA extraction.

### Soil properties, tree biomass, and climate factors

Soil pH was measured in a soil:water (1:2.5, w/v) solution using a glass electrode. Soil moisture was determined gravimetrically by drying fresh soil samples at 105°C for 24 h and calculating the weight loss. Soil total carbon and total nitrogen (TN) were accurately assayed using an Elementar Vario EL III (Elementar Analysensysteme GmbH, Langenselbold, Germany). Soil dissolved organic carbon (DOC) was measured using a total organic carbon analyzer (TOC-VCPH; Shimadzu, Kyoto, Japan). Soil ammonium nitrogen (NH_4_^+^-N) and nitrate nitrogen (NO_3_^−^-N) contents were extracted using a 2.0 M KCl solution and were then measured using a continuous flow analyzer (SAN11; Skalar, Breda, Netherlands). Soil AP content was quantified using the Mehlich-3 method. The soil physicochemical data have been described previously (36, 37). The diameter at breast height (DBH) of the Chinese fir tree was measured using a tape measure or vernier caliper in August 2022 (DBH1, D_1_) and August 2023 (DBH2, D_2_), respectively. Subsequently, we calculated the biomass (Basal area, the average basal area of Chinese fir trees measured in 2022 and 2023; and Increment, the difference in basal area between 2022 and 2023) of the tree. Basal area = [π(D_1_/2)^2^ + π(D_2_/2)^2^]/2; Increment = π(D_2_/2)^2^ − π(D_1_/2)^2^ (38). Climatic factors of mean annual precipitation (MAP) and mean annual temperature (MAT) were gained from the WorldClim database (https://www.worldclim.org).

### DNA extraction, polymerase chain reaction (PCR), and sequencing

Root samples were thoroughly cleaned with water to remove soil residues and were then washed three times with sterile water. Post freeze-drying using liquid nitrogen, the roots were homogenized using a sterilized mortar and pestle (33). DNA was extracted from 250 mg of each root and rhizospheric soil sample using the PowerSoil DNA isolation kit (MoBio Laboratories Inc., Carlsbad, CS, USA), following the manufacturer’s instructions. The DNA concentrations were measured using a NanoDrop 1000 Spectrophotometer (Thermo Scientific, Wilmington, DE, USA).

The V4 region of the 16S rRNA gene was amplified using the primers 515F and 806R (39). The thermocycling conditions included initial denaturation at 94°C for 5 min, followed by 35 cycles of denaturation at 94°C for 50 s, annealing at 52°C for 1 min, and extension at 68°C for 1 min. A barcode (12 bases) was added to the 5′ side of 806R to indicate the sample origin. Fungal-specific primers 5.8S-Fun and ITS4-Fun (40), linked with a 12-base barcode, were used in this study. The thermocycling conditions included initial denaturation at 95°C for 3 min, followed by 35 cycles of denaturation at 94°C for 30 s, annealing at 58°C for 40 s, and extension at 72°C for 1 min. Each DNA sample was amplified in triplicate. Subsequently, the PCR products were purified using the Wizard SV Gel and PCR Clean-Up System (Promega, Madison, WI, USA) and were pooled with equimolar amounts from each sample. After quantifying the DNA concentrations using a TBS 380 fluorescence spectrophotometer (Promega, Madison, WI, USA), the purified PCR products were mixed in equimolar amounts and sequenced on an Illumina NovaSeq sequencer (Illumina, San Diego, CA, USA).

### Bioinformatics analyses

Raw FASTQ files were de-multiplexed using an in-house perl script and then quality-filtered using fastp version 0.19.6 (41) and merged using FLASH version 1.2.11 (42). Low-quality reads were removed, including those missing valid primer/barcode sequences, those containing ambiguous bases, or those with an average quality score lower than 20. Based on the overlap relationships between paired-end reads, the paired reads were merged into a single sequence with a minimum overlap length of 10 base pairs. A maximum mismatch ratio of 0.2 was allowed for the merged sequences in the overlap region, and those that did not meet this criterion were filtered out. Subsequently, the samples were distinguished based on the barcodes and primers at both ends of the sequences, and the sequence orientation was adjusted accordingly. The allowed number of mismatches for the barcode was 0, whereas the maximum number of primer mismatches allowed was 2. Chimera sequences were checked using USEARCH version 8.0 software (43). High-quality sequences were subjected to de-singleton and de-replication and clustered into operational taxonomic units (OTUs) at a 97% identity threshold using the UPARSE pipeline (44) and USEARCH. The representative sequences of each OTU were selected using the “get.oturep” command and identified using the SINTAX algorithm against the Ribosomal Database Project (RDP) (rdp_16s_v16) and UNITE database with a confidence threshold of 65% (45), excluding chloroplast DNA sequences. To eliminate the potential effects of uneven sequence depths across samples on bacterial and fungal community analyses, the number of sequences per sample was rarefied to the smallest sample size using the “sub.sample” command in Mothur 1.32.2 (46). The resulting bacterial and fungal community matrices were used to assess *α*- and *β*-diversity, i.e., variations in microbial communities.

### Statistical analyses

Quantitative data were statistically analyzed using SPSS (version 26.0; SPSS Inc., Chicago, IL, USA) and R (version 4.3.2). Rarefaction curves were calculated using the “specaccum” function in the “vegan” package. *α*-diversity indices, including OTU richness and Shannon index, were computed using the same package (47). The pairwise comparisons of *α*-diversity among plant compartments and sites were performed using one-way analysis of variance (ANOVA) or Kruskal–Wallis test (variance heterogeneity) to determine potential significant differences at a *P*-value < 0.05. Two-way ANOVA (SPSS) was also used to demonstrate the effects of compartment and site on bacterial and fungal *α-*diversity.

Permutational multivariate ANOVA (PERMANOVA) was performed based on distance matrices using the “adonis” function in the “vegan” package with 999 permutations (48) to evaluate the effects of plant compartments and sites on bacterial and fungal *β-*diversity. Bacterial and fungal community compositions were ordinated using non-metric multidimensional scaling (NMDS) with dissimilarity matrices, with the “metaMDS” function of the “vegan” package. The correlation heatmap was used to demonstrate correlations between the biotic and abiotic factors using the “Pearson” method in the “corrplot” package. The linear regression of the relationships between plant biomass parameters (basal area and growth increment) and bacterial and fungal *α*- and *β-*diversity (richness and NMDS1) in both compartments was visualized using the Origin 2024 software.

Random forest analysis was performed to identify the most critical factors affecting basal area and growth increment in Chinese fir trees using the rfPermute function in the “rfPermute” package. Finally, we performed structural equation modeling (SEM) (49) to explore the direct and indirect pathways through which soil properties, microbial diversity, and community composition impacted the growth status (tree biomass and growth increment) of Chinese fir trees. We first considered a hypothesized conceptual model that included all reasonable pathways. Subsequently, we sequentially eliminated non-significant pathways, unless they were biologically informative, or added pathways based on the residual correlations. The procedure was repeated until the model exhibited sufficient fitting with the *P-*values of the *χ*^2^ test larger than 0.05, indicating that the predicted model and observed data were not significantly different, and the root mean square error of approximation (RMSEA) was less than 0.08. The SEM-related analysis was performed using the “lavaan” package (50).

## RESULTS

### Molecular identification

After quality control, 11,772,749 and 12,769,883 sequences were identified and classified into 24,934 bacterial OTUs and 10,919 fungal OTUs derived from high-quality sequences. We excluded 102 archaeal and 233 cyanobacterial OTUs from the 24,934 bacterial OTUs. Subsequently, we performed normalization using 10,005 bacterial sequences (10,005–184,238 sequences among 108 rhizospheric soil and 108 root samples) and 12,452 fungal sequences (12,452–161,613 sequences among 108 rhizospheric soil and 104 root samples, as four root samples were excluded because of PCR failure). Finally, 24,599 and 10,919 bacterial and fungal OTUs, respectively, were obtained for the downstream analyzes.

These bacterial and fungal OTUs were primarily categorized into 14 bacterial phyla and six fungal phyla, respectively (Fig. S1). The bacterial OTUs exhibited a diverse taxonomic distribution, encompassing 24 phyla. The dominant phyla were Proteobacteria, Acidobacteria, and Actinobacteria (Fig. S1a). Higher abundances of Proteobacteria and Acidobacteria were detected in two compartments (rhizospheric soil and root). The relative abundances of the dominant bacterial phyla were significantly different between rhizospheric soil and root samples. For example, the abundance of Acidobacteria was significantly higher in rhizospheric soil than in root samples (Fig. S1). The identified fungal OTUs were found to be distributed in 12 phyla. Ascomycota (63.24%) and Basidiomycota (22.16%) were the most abundant fungal phyla (Fig. S1b). Similarly, the compartment factor significantly affected the relative abundances of Ascomycota and Basidiomycota. For instance, Ascomycota accounted for 67.26% in rhizospheric soil, but 59.21% in root (Fig. S1b). The rarefaction curves for both compartments tended to reach an asymptote, indicating that the majority of the distinct bacterial and fungal OTUs had been recovered (Fig. S2). Furthermore, variations in the relative abundances of these phyla were observed across different sites.

### Bacterial and fungal *α*-diversity

**Fig 1:**
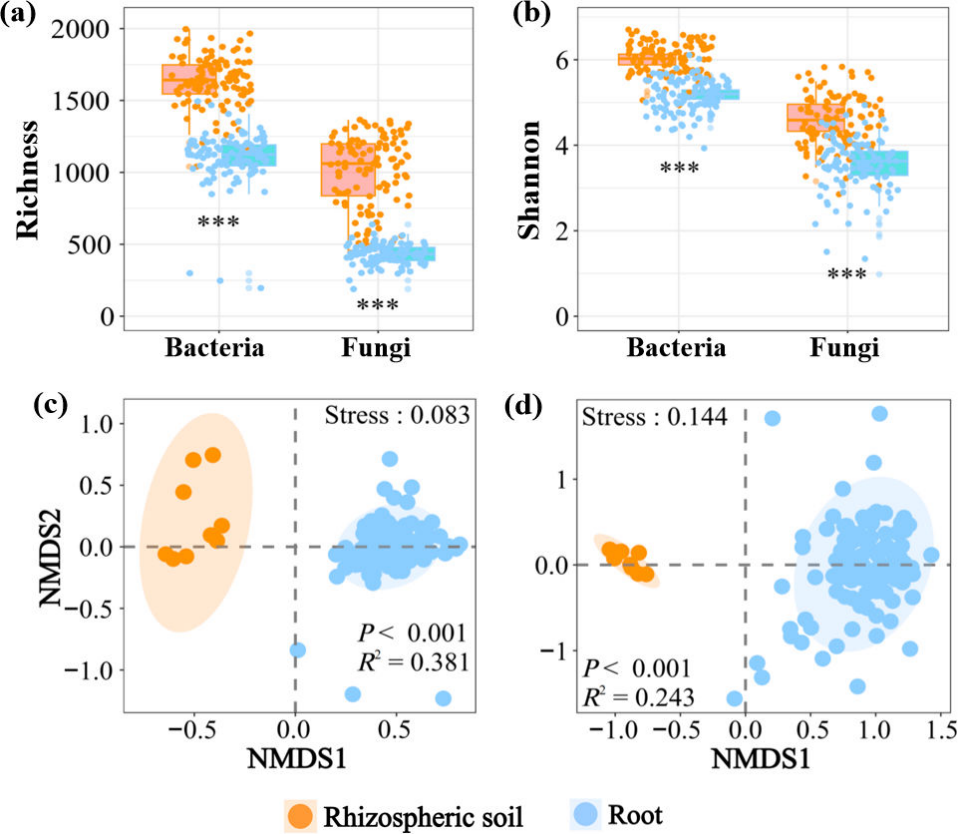
Box plots showing the distribution of bacterial and fungal operational taxonomic unit richness (a) and the Shannon index (b) of the rhizospheric soil- and root-associated samples. The center line of each box plot indicates the mean, the lower and upper hinges indicate the standard deviation around the mean, and each whisker corresponds to the minimum and maximum values, respectively. Asterisks indicate the levels of significance (*P < 0.05; **P < 0.01; and ***P < 0.001). Non-metric multidimensional scaling (NMDS) ordinations of the bacterial (c) and fungal (d) community composition in rhizospheric soil and root compartments. The elliptic shadows indicate 95% confidence intervals around the centroids of rhizospheric soil- and root-associated bacterial and fungal communities. The stress values are 0.085 and 0.144 for bacterial and fungal two-dimensional NMDS analyses, respectively. The results of the permutational multivariate analysis of variance, including *R*^2^ and *P-*values, depict significant differences in bacterial and fungal community composition between the rhizospheric soil- and root-associated samples.

In this study, *α*-diversity was defined based on OTU richness and the Shannon index. Both plant compartment and site factors exerted significant effects on rhizospheric soil- and root-associated bacterial and fungal *α*-diversity (*P* < 0.001, Table 1). Significantly higher bacterial and fungal OTU richness and Shannon indices were detected in rhizospheric soil than in tree root samples, regardless of plantation sites (Fig. 1a and b; Fig. S3). In the rhizospheric soil samples, the highest bacterial and fungal OTU richness was observed at the JS and XY sites, respectively (Fig. S3a and b); however, in the tree root samples, the highest bacterial and fungal OTU richness was observed at the XY and WYS sites, respectively, whereas the lowest OTU richness was detected at the JS site for both bacteria and fungi (Fig. S3a and b). The Shannon indices showed a similar pattern across nine plantation sites (Fig. S3c and d).

**TABLE 1:** Results for the two-way analysis of variance showing the effects of plant compartments and sites on the operational taxonomic unit richness and Shannon indices of bacterial and fungal communities^*a*^

Variation source	Bacteria				Fungi			
	Richness		Shannon		Richness		Shannon	
	**F**	***P***	**F**	***P***	**F**	***P***	**F**	***P***
Compartment	723	**<0.001**	1103	**<0.001**	1355	**<0.001**	283	**<0.001**
Site	3.699	**<0.001**	3.793	**<0.001**	24.259	**<0.001**	4.339	**<0.001**
Compartment × site	11.875	**<0.001**	7.610	**<0.001**	19.604	**<0.001**	5.225	**<0.001**

^*a*^ Compartment implies two types of samples, i.e., rhizospheric soil-associated and root-associated; Site denotes nine different Chinese fir plantation sites. P-value in bold presents the significance level.

### Bacterial and fungal *β*-diversity

**Fig 2:**
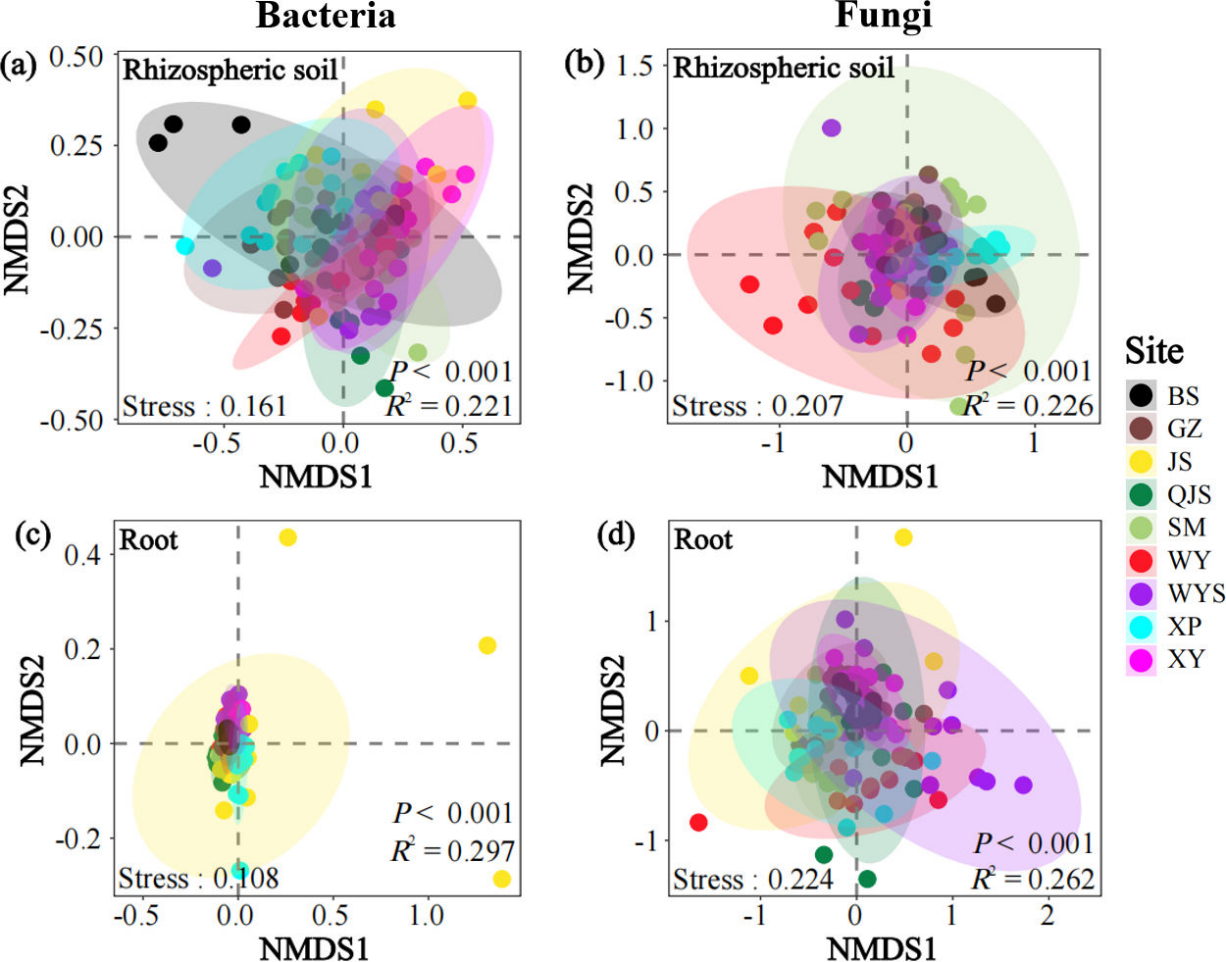
Non-metric multidimensional scaling (NMDS) ordinations of the bacterial (a, c) and fungal (b, d) community composition in rhizospheric soil-associated (a, b) and root-associated (c, d) compartments across nine Chinese fir stands. Elliptic shadows indicate 95% confidence intervals around the centroids of the different sites. *R*^2^ and *P*-values represent the results of a permutational multivariate analysis of variance (adonis). Abbreviations: BS, Baisha State-owned Forest Farm in Shanghang County; GZ, Guanzhuang State-owned Forest Farm in Sha County; JS, Jinshan State-owned Forest Farm in Hua’an County; QJS, Qiujiashan State-owned Forest Farm in Liancheng County; SM, Fujian Sanming Forest Ecosystem National Observation and Research Station in Sanming City; WY, Wuyi State-owned Forest Farm in Zhangping City; WYS, Wuyishan National Park in Wuyishan City; XP, Xiapu State-owned Forest Farm in Ningde City; XY, Xiayang State-owned Forest Farm in Nanping City.

PERMANOVA results revealed significant differences in the composition of bacterial (*R*^2^ = 0.381; *P* < 0.001) and fungal (*R*^2^ = 0.243; *P* < 0.001) communities between the rhizospheric soil and root compartments of Chinese fir trees, supported by NMDS ordination visualization graphs (Fig. 1c and d). Moreover, site factor also exerted significant effects on bacterial and fungal community composition (*P* < 0.001 in all cases) regardless of rhizospheric soil and tree root samples (Fig. 2). Unlike the rhizospheric soil, the bacterial communities of the root-associated compartment were more convergent in most sites except for the JS site (Fig. 2c). Both rhizospheric soil- and root-associated fungal communities varied clearly across nine plantation sites (Fig. 2b and d).

### Effects of soil properties, climate, and microbial factors on tree growth

**Fig 3:**
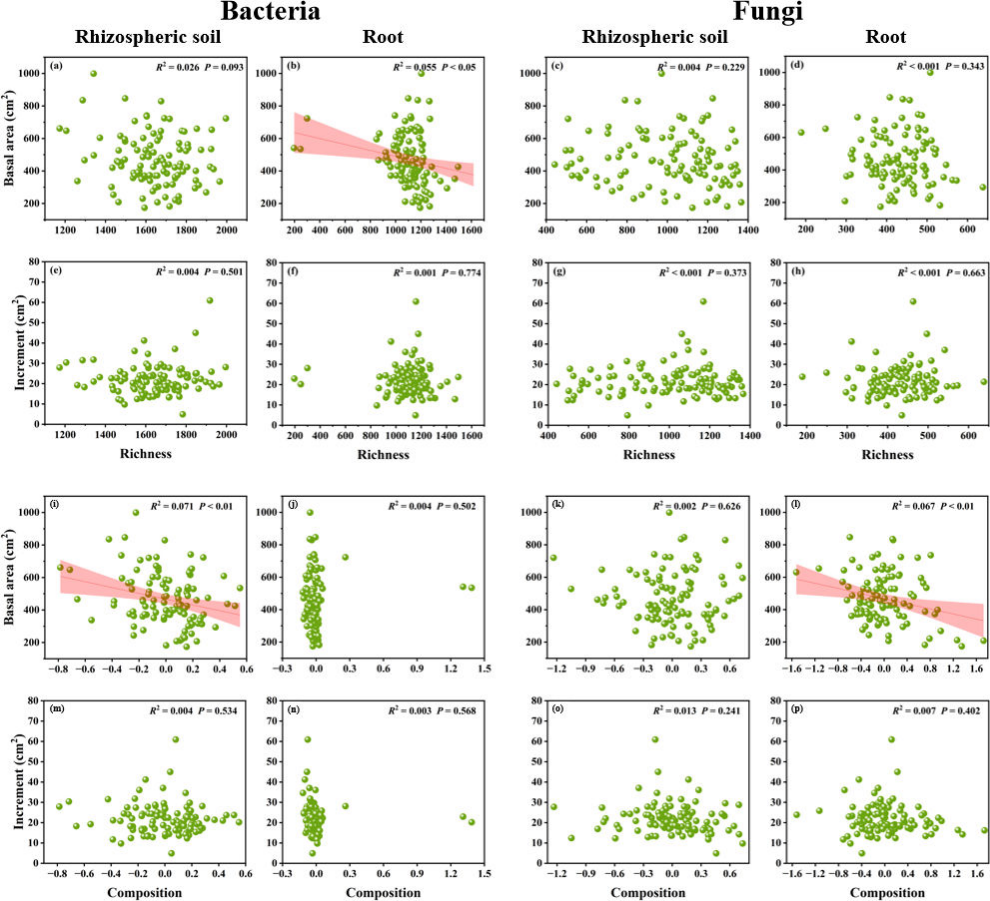
Relationships between the basal area (a, b, c, d) and one-year growth increment (e, f, g, h) of Chinese fir trees and bacterial or fungal operational taxonomic unit richness; and between the basal area (i, j, k, l) and 1-year growth increment (m, n, o, p) of these trees and bacterial or fungal *β*-diversity (community composition).

**Fig 4:**
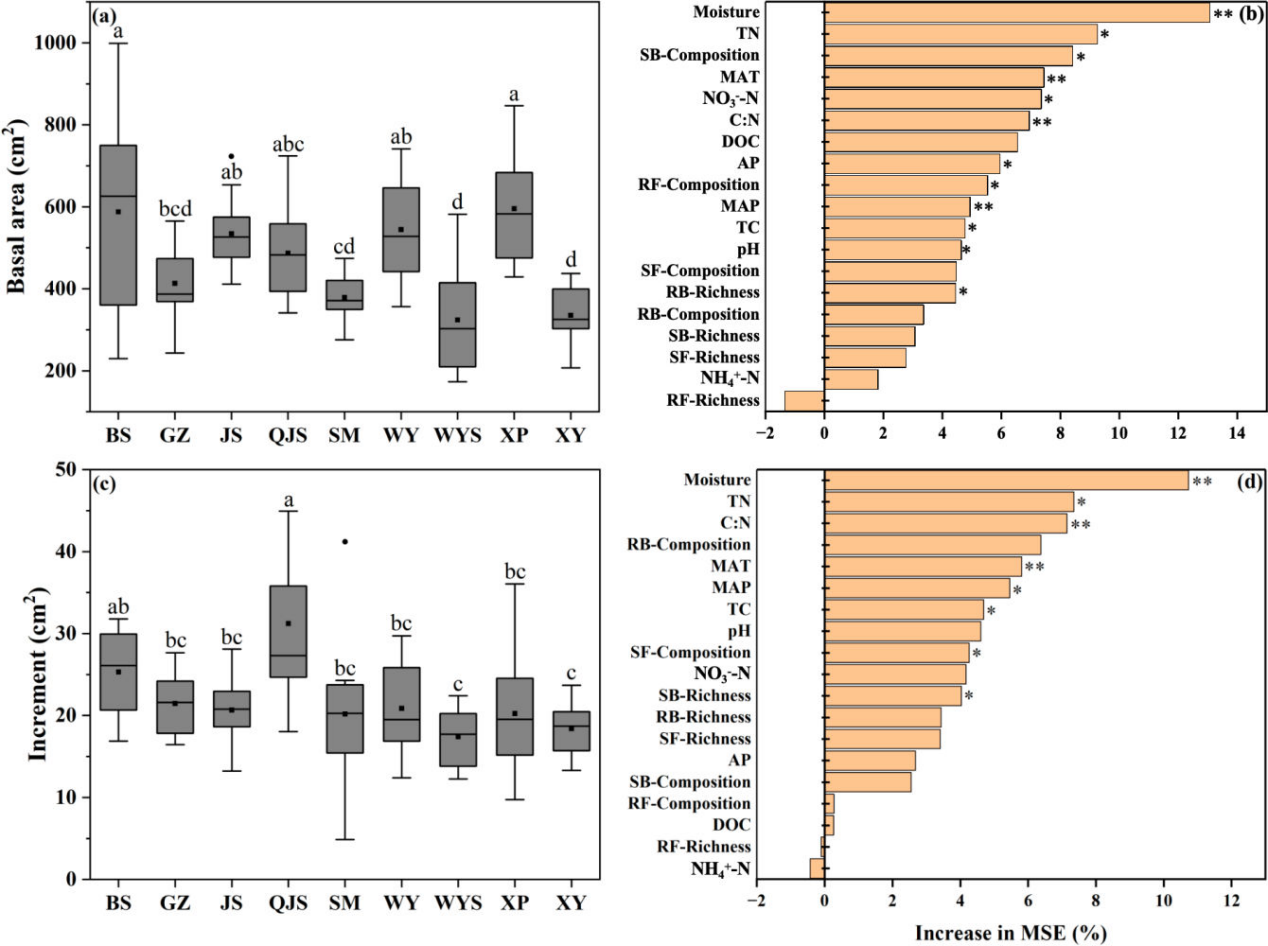
Comparison of the basal area (a) and 1-year growth increment (c) in trees across nine Chinese fir plantation sites. Lowercase letters indicated the significant difference in basal area or growth increment among different sites. Random forest analysis reveals the importance of biotic and abiotic factors affecting the basal area (b) and growth increment (d) in Chinese fir trees. Asterisk symbols indicate significance at *P* < 0.05 (*) and *P* < 0.01 (**) levels. Abbreviations: SB-Composition, bacterial community composition detected in rhizospheric soil using the first axis of non-metric multidimensional scaling (NMDS) ordination (NMDS1) as a proxy; AP, soil available phosphorus; MAT, mean annual temperature; MAP, mean annual precipitation; RB-Richness, bacterial richness detected in tree roots; NO_3_^−^-N, soil nitrate nitrogen content; TC, soil total carbon; DOC, soil dissolved organic carbon; SF-Composition, fungal community composition detected in rhizospheric soil using NMDS1 as a proxy; RB-Composition, bacterial community composition detected in tree roots using NMDS1 as a proxy; TN, soil total nitrogen; RF-Composition, fungal community composition detected in tree roots using NMDS1 as a proxy; SF-Richness, fungal richness detected in rhizospheric soil; SB-Richness, bacterial richness detected in rhizospheric soil; NH_4_^+^-N, soil ammonium nitrogen content; RF-Richness, fungal richness detected in tree roots. BS, Baisha State-owned Forest Farm in Shanghang County; GZ, Guanzhuang State-owned Forest Farm in Sha County; JS, Jinshan State-owned Forest Farm in Hua’an County; QJS, Qiujiashan State-owned Forest Farm in Liancheng County; SM, Fujian Sanming Forest Ecosystem National Observation and Research Station in Sanming City; WY, Wuyi State-owned Forest Farm in Zhangping City; WYS, Wuyishan National Park in Wuyishan City; XP, Xiapu State-owned Forest Farm in Ningde City; XY, Xiayang State-owned Forest Farm in Nanping City.

We observed that basal area was affected by soil properties, e.g., moisture and TN, climate factors, i.e., MAT and MAP, and microbial factors, including root-associated bacterial richness (RB-Richness), soil-associated bacterial composition (SB-Composition), and root-associated fungal composition (RF-Composition). The basal area of trees correlated negatively with bacterial diversity (RB-Richness) and community composition (SB-Composition) and fungal community composition (RF-Composition) (Fig. S4). These results were further supported by the linear regression analysis results, which revealed that RB-Richness, SB-Composition, and RF-Composition were negatively associated with basal area (Fig. 3b, i, and l). The random forest analysis further revealed that soil moisture was the most critical factor to predict the growth status (basal area and increment) of Chinese fir trees (Fig. 4).

**Fig 5:**
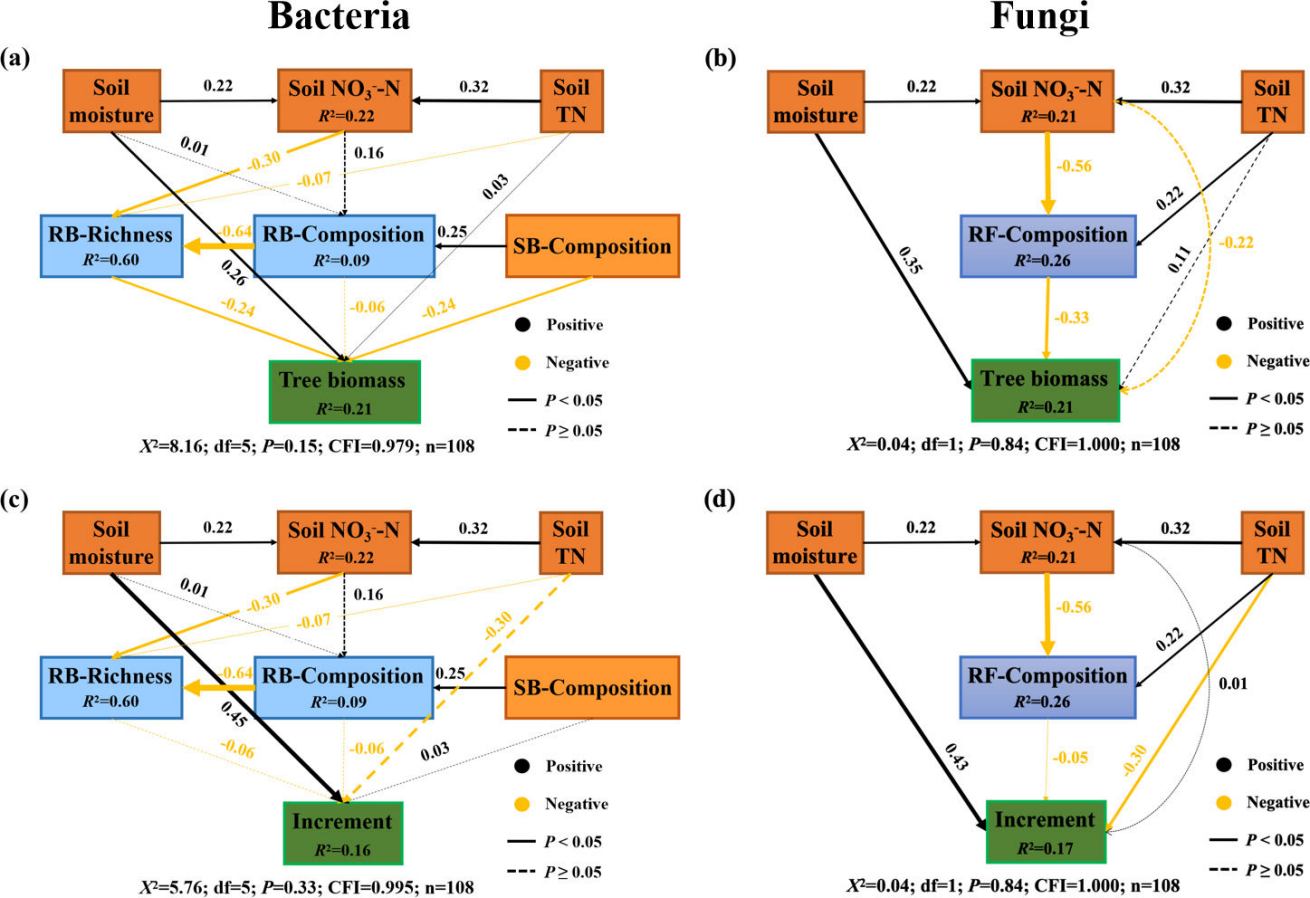
Structural equation models (SEMs) showing the causal relationships between soil properties, microbial diversity, and community composition, which was assessed using the first axis of non-metric multidimensional scaling (NMDS) ordination (NMDS1) as a proxy, and tree growth based on tree biomass (a, b) and growth increment (c, d). The black and yellow arrows indicate positive and negative relationships, respectively. The solid and dashed lines indicate significant (*P* < 0.05) and non-significant (*P* ≥ 0.05) pathways, respectively, whereas the strength of the causal effect is indicated by the standard path coefficients presented close to each line. The width of the solid lines indicates the strength of the causal effect, based on the standard path coefficients presented close to each line. *R*^2^ represented the proportion of variance explained for each variable. The final models fitted the data well: maximum likelihood, *χ*² =0.04-4.60; comparative fit index (CFI) ≥0.985; and all *P*-values > 0.05. Abbreviations: Soil NO_3_^−^-N, soil nitrate nitrogen content; Soil TN, soil total nitrogen; RB-Richness, bacterial richness detected in tree root; RB-Composition, bacterial community composition detected in tree root using NMDS1 as a proxy; SB-Composition, bacterial community composition detected in rhizospheric soil using NMDS1 as a proxy; RF-Composition, fungal community composition detected in tree root using NMDS1 as a proxy; Tree biomass, basal area of trees.

To further distinguish the direct and indirect effects of environmental drivers, microbial diversity, and community composition on the growth of Chinese fir trees, SEM analyses were performed with presumed relationships between the subsets of least-correlated plant, soil, and microbial variables (Fig. 5). Soil NO_3_^−^-N content was positively affected by soil moisture and TN content (*P* < 0.05). RB-Richness and RF-Composition correlated significantly negatively with the soil NO_3_^−^-N content. SB-Composition directly and positively affected RB-Composition, whereas it directly and negatively affected tree biomass (*P* < 0.05). RF-Composition directly and negatively affected tree biomass (*P* < 0.05); however, no significant impact was observed on tree growth increment. In comparison, among the variables which directly contribute to tree biomass, only the paths of soil moisture, RB-Richness, SB-Composition, and RF-Composition were significant (*P* < 0.05). Overall, soil NO_3_^−^-N and TN contents could also impact tree biomass by directly impacting RB-Richness and RF-Composition; however, they did not exert a direct effect on tree growth increment. Soil moisture played a direct and critical role in shaping tree biomass, consistent with the results of Pearson’s correlation and random forest analyses (Fig. S4; Fig. 4).

## DISCUSSION

### Microbial *α*- and *β*-diversity was impacted by plant compartment and site

Microorganisms inhabiting the roots and surrounding soil are of paramount importance for plant health, as they enhance nutrient availability, promote the production of hormones, and aid plants in resisting biotic and abiotic stresses (51–53). Our investigation of bacterial and fungal communities in the rhizospheric soil and root compartments of Chinese fir plantations revealed significant differences in their *α*- and *β*-diversity. Both compartment and site exerted significant effects on bacterial and fungal *α*- and *β*-diversity (Table 1). Notably, the rhizospheric soil exhibited significantly higher bacterial and fungal *α*-diversity compared to the root compartment (Fig. 1a and b). Previous studies also demonstrated that the soil compartment (of which rhizospheric soil is a part) harbored a higher microbial *α*-diversity than did the other compartments (32, 34, 54). However, contrasting results have also been reported, with higher *α*-diversity observed in the phyllosphere (33) and root endosphere (55) compartments. In the present study, the bacterial and fungal diversity in root samples was lower than in rhizospheric soil samples, which could be attributed to the following reasons. First, root samples were subject to physical filtering, i.e., root hair barrier, under the host selection effect, which shapes bacterial and fungal communities (56, 57). Second, root exudates, i.e., sugars, amino acids, and organic acids, may have benefited microbial taxa that could utilize them, triggering chemotactic responses from microbes. Third, root exudates containing antimicrobial compounds could reduce the growth of susceptible microbes (58–61). Finally, the greater heterogeneity of the rhizospheric soil likely results in higher bacterial and fungal diversity than in the relatively homogeneous root compartments.

Previous studies have consistently shown that bacterial and fungal community compositions are extremely sensitive to environmental variations (62, 63). Therefore, it was speculated that different compartments within the soil–plant system would have significant impacts on the *β*-diversity of these microbial communities. For example, at the phylum level, Proteobacteria, Acidobacteria, and Actinobacteria were prominent in both compartments (Fig. S1), which is consistent with the results of previous studies that these phyla are abundant in soil (64) and roots (62), implying that these phyla are likely the bacterial groups closely associated with Chinese fir identity (33). Furthermore, a few members of the Proteobacteria and Acidobacteria phyla are known to participate in nitrogen fixation and cycling (65, 66), highlighting their potential roles in promoting plant growth and biocontrol, vital for maintaining the productivity and resilience of Chinese fir plantation ecosystems. Despite extensive research on microbial communities in belowground compartments, i.e., rhizospheric soil and roots, the differences in microbial *β*-diversity between these two compartments in Chinese fir plantations remain to be elucidated. Our study bridges this gap, revealing a remarkable discrepancy between the root and rhizospheric soil. Specifically, the rhizospheric soil compartment exhibited greater variability and divergence than in the root compartment, indicating a strong heterogeneity of the rhizospheric soil across the nine sites. Alternatively, factors shaping microbial communities in the rhizospheric soil and root compartments of Chinese fir plantations may differ significantly. This speculation was supported by the finding that root-associated bacterial and fungal community compositions were significantly shaped by soil properties, such as soil TN and NO_3_^−^-N contents (Fig. S4).

### Drivers mediating the growth of Chinese fir trees

Modern forestry management prioritizes ecological considerations, such as carbon sequestration and biodiversity maintenance, over timber production (67, 68). Measuring changes in forest biomass is critical for establishing forest management policies (69). Therefore, understanding how biotic and abiotic factors affect the biomass of Chinese fir trees and the underlying mechanisms is a critical issue in host plant and microbiology research. By examining the dynamic changes of the biomass of Chinese fir plantations across nine sites, this study provided explicit evidence that RB-Richness, SB-Composition, and RF-Composition influenced the biomass of Chinese fir trees. In addition, by examining the interactive effects of environment variables, microbial diversity, and community composition, this study demonstrated that soil TN and NO_3_^−^-N played predominant roles in shaping RB-Richness and RF-Composition, and, to our knowledge, it is the first study to demonstrate that soil TN and NO_3_^−^-N play predominant roles in shaping the biomass of Chinese fir via altering both bacterial diversity and fungal community composition in tree root (Fig. 5).

Moreover, our study has also revealed that soil moisture and TN were also the key factors influencing the increment of Chinese fir (Fig. 5). Soil nitrogen and water contents are tightly coupled to the growth of plants. Previous studies have reported that increased soil water content and nitrogen supply can lead to a significant increase in plant growth and biomass production (70, 71). Water affects all plant growth processes, including nutrient absorption, translocation, photosynthesis, respiration, transpiration, cell division, and metabolic reactions within the plant system and root zone. However, the availability of soil water to plants differs significantly based on soil, plant, and environmental conditions (72). Additionally, studies have shown that trees in plots with favorable site conditions exhibit higher aboveground biomass growth than those growing under poorer conditions (73). When nutrient requirements are unmet during rapid tree growth, tree biomass suffers a negative impact (74). This pattern was also observed in our study, wherein we investigated the effects of variations in soil properties on tree growth and biomass. Notably, the biomass of Chinese fir trees varied across different sites, and soil moisture and TN contents were the key factors significantly shaping the growth (increment) of these trees (Fig. 4 and 5d). These differences in biomass could be attributed to the strategies adopted by trees to maximize light, nutrient, and water capture for survival under varying site conditions (75, 76). Notably, our findings revealed that site quality significantly impacted biomass, adding complexity to its dynamics (77). Therefore, our study highlights that soil moisture and N content jointly shape the biomass of Chinese fir trees.

Chinese fir forms a close symbiotic relationship with AM fungi, which significantly promotes nutrient absorption and transfer, thus enhancing the growth and biomass accumulation of the tree. In this study, we detected significant negative correlations between tree biomass (basal area) and rhizospheric soil-associated bacterial community composition, root-associated bacterial richness, and root-associated fungal community composition (Fig. 3 and 5). Similarly, it has been reported that fungal community composition correlates significantly negatively with tree growth in European temperate forests (23). Our intriguing finding necessitates a deeper exploration of its potential causes. First, certain bacteria, as well as saprotrophic and mycorrhizal fungi, can outcompete trees in nutrient uptake. For example, saprotrophic fungi may sequester nutrients or form inaccessible complexes, whereas mycorrhizal fungi can adopt aggressive uptake strategies in low-nutrient soils (78). Second, certain pathogenic fungi can disrupt plant physiological processes, causing root cell death and triggering energy-consuming defense responses (79, 80), thereby inhibiting growth. Finally, pathogenic fungi can invade roots, and some can form dense mycelial mats that restrict root growth (80). Root-architecture and histological studies could be adopted to demonstrate root damage and growth changes. Additionally, plants select root-colonizing microbes, favoring symbiotic species (17, 81); thus, any shifts in microbial community composition away from preferred partners would disrupt tree growth. Exploring these pathways and performing the relevant experiments would help enhance our understanding of the plant–microbe relationship, aiding forest management and conservation.

### Practical implications for forest management

Considering the crucial effects of microbial communities on plant growth and biomass, certain microbial-centered strategies could be adopted in forest management practice. For example, plant growth and fitness could be improved by inoculating plants with beneficial soil microbes, such as nitrogen-fixing bacteria and mycorrhizal fungal communities (82–84). Notably, to obtain temporal data across multiple growing seasons and improve the detection of rare or unsequenced microbes, it will be helpful to determine the causal impacts of microbial communities on plants’ fitness. Theoretically, after testing the effects of various biofertilizers with specific microbial strains over multiple seasons on tree growth and microbial community structure, we could assess the efficacy of composite microbial populations and uncover the underlying microbial mechanisms for promoting tree growth (85–87). In practice, for the management of planted forests, we can adopt controlled experiments related to *in situ* microbiome manipulation, microbial inoculants, and synthetic microbial community design to provide key evidence to confirm the causal roles of microbial communities in tree growth and forest productivity.

### Conclusions

Understanding the variations of tree biomass is essential for accurately estimating forest productivity and carbon storage. The results obtained in this study revealed the biomass and growth of Chinese fir trees, as well as the effects of geographical locations, microbial diversity, and community composition on the biomass and growth parameters of these trees. Both plant compartment and site factors exhibited significant effects on microbial community structures, with higher soil-associated bacterial and fungal *α*-diversity than root-associated bacterial and fungal *α*-diversity. Soil-associated bacterial community composition exhibited a considerable impact on the biomass of Chinese fir trees, whereas both root-associated bacterial diversity and fungal community composition exerted a relatively greater impact on tree biomass. Stronger correlations were observed between soil factors and bacterial richness and fungal community composition in root compartments than between soil factors and bacterial richness and fungal community composition in soil compartments, with soil TN and NO_3_^−^-N contents emerging as two key drivers of those associations. Our findings enhance the understanding of the roles of microbial diversity and community composition in shaping the growth and biomass of fir trees. Although additional evidence from other ecosystems would be needed to support these findings, the results obtained in this study confirmed that root-associated microbiomes can be crucial for promoting timber productivity in Chinese fir plantations. Overall, although the results obtained in this study provide valuable insights, several limitations exist, including the lack of temporal data across multiple growing seasons or the reliance on specific sequencing techniques that may miss rare or unsequenced microbes. Therefore, in future studies, it would be essential to incorporate multiple sampling designs spanning multiple years or growing seasons to capture temporal fluctuations and patterns. Additionally, employing a combination of complementary sequencing technologies (e.g., metagenomics and metatranscriptomics) could enhance the detection of rare taxa, providing a more comprehensive understanding of microbial community structures and functions.

## SUPPLEMENTAL MATERIAL

Supplemental figures msphere.00450-25-s0001.docx Fig. S1 to S4.

ASM does not own the copyrights to Supplemental Material that may be linked to, or accessed through, an article. The authors have granted ASM a non-exclusive, world-wide license to publish the Supplemental Material files. Please contact the corresponding author directly for reuse.
