# Supplementary material for: Regional-scale biogeographical patterns of soil- and root-associated microbial communities across nine planted Chinese fir forests
Source: mSphere. 2025 Jul 31;10(8):e00450-25. doi: 10.1128/msphere.00450-25 (PMC12379592; doi:10.1128/msphere.00450-25)
Supplement: Supplemental figures — Fig. S1 to S4. [file msphere.00450-25-s0001.docx]

***Title Page***

# Supplementary information

**Title:** Regional-scale biogeographical patterns of soil- and root-associated microbial community across nine planted Chinese fir forests

**Authors:** Feihua Zhou^1^, Hanshuo Zhang^1^, Wen Zhong^1^, Hao Yang^1^, Luhong Zhou^1,2^, Zhi-Jie Yang^3^, Yalin Hu^4^, Yong Zheng^1,2,^*

**Affiliations:**

^1^ Key Laboratory for Humid Subtropical Eco-geographical Processes of the Ministry of Education, Fujian Normal University, Fuzhou 350117, China.

^2^ Shanghang Mountain Forest Carbon Sink Observation and Research Station of Fujian Province, Fujian Normal University, Longyan 364205, China.

^3^ Fujian Sanming Forest Ecosystem National Observation and Research Station, Fujian Normal University, Sanming 365002, China.

^4^ Forest Ecology Stable Isotope Center, College of JunCao Science and Ecology, Fujian Agriculture and Forestry University, Fuzhou, 350002, China.

*Author for correspondence: Dr. Yong Zheng

*Tel.*: +86 (0)591 8346 5214; *Fax*: +86 (0)591 8346 5397; *E-mail*: zhengy@fjnu.edu.cn (Y.Z.)

# Running title: Biogeographical patterns of soil- and root-associated microbial communities

# Figure legend

**Figure S1** Relative abundance of bacterial (**a**) and fungal (**b**) taxa at the phylum level in rhizospheric soil and root compartments across nine Chinese fir stands. Taxa in the “Others” are the relative abundance of the bacterial and fungal phyla less than top 10 phyla. Abbreviations: BS, Baisha State-owned Forest Farm in Shanghang County; GZ, Guanzhuang State-owned Forest Farm in Sha County; JS, Jinshan State-owned Forest Farm in Hua’an County; QJS, Qiujiashan State-owned Forest Farm in Liancheng County; SM, Fujian Sanming Forest Ecosystem National Observation and Research Station in Sanming City; WY, Wuyi State-owned Forest Farm in Zhangping City; WYS, Wuyishan National Park in Wuyishan City; XP, Xiapu State-owned Forest Farm in Ningde City; XY, Xiayang State-owned Forest Farm in Nanping City.

**Figure S2** Rarefaction curves for the observed bacterial (**a**) and fungal (**b**) OTUs among two compartments of Chinese fir stands. 108 rhizospheric soil samples of bacteria (**c**) and fungi (**d**) among nine Chinese fir stands, 108 root samples of bacteria (**e**) and 104 root samples of fungi (**f**) among nine Chinese fir stands. Abbreviations: BS, Baisha State-owned Forest Farm in Shanghang County; GZ, Guanzhuang State-owned Forest Farm in Sha County; JS, Jinshan State-owned Forest Farm in Hua’an County; QJS, Qiujiashan State-owned Forest Farm in Liancheng County; SM, Fujian Sanming Forest Ecosystem National Observation and Research Station in Sanming City; WY, Wuyi State-owned Forest Farm in Zhangping City; WYS, Wuyishan National Park in Wuyishan City; XP, Xiapu State-owned Forest Farm in Ningde City; XY, Xiayang State-owned Forest Farm in Nanping City.

**Figure S3** Comparison of rhizospheric soil- and root-associated microbial OTU richness (**a**, bacteria; **b**, fungi) and Shannon index (**c**, bacteria; **d**, fungi) across nine Chinese fir stands. Boxplot centre line indicates the mean, lower and upper hinges the standard deviation around the mean and each whisker correspond to the minimum and maximum values, respectively. Different uppercase and lowercase letters indicate significant differences in OTU richness and Shannon index for rhizospheric soil- and root-associated samples, respectively, across nine plantation locations (P < 0.05). Asterisks indicate levels of significance (**P* <0.05; ***P* < 0.01; ****P* < 0.001). Abbreviations: BS, Baisha State-owned Forest Farm in Shanghang County; GZ, Guanzhuang State-owned Forest Farm in Sha County; JS, Jinshan State-owned Forest Farm in Hua’an County; QJS, Qiujiashan State-owned Forest Farm in Liancheng County; SM, Fujian Sanming Forest Ecosystem National Observation and Research Station in Sanming City; WY, Wuyi State-owned Forest Farm in Zhangping City; WYS, Wuyishan National Park in Wuyishan City; XP, Xiapu State-owned Forest Farm in Ningde City; XY, Xiayang State-owned Forest Farm in Nanping City.

**Figure S4** Correlation heat map showing the correlations among the biotic and abiotic factors. Pairwise comparisons of factors are displayed, with a color gradient denoting Pearson’s correlation coefficient. Abbreviations: DOC, soil dissolved organic carbon; TC, soil total carbon; TN, soil total nitrogen; NO_3_^–^-N, soil nitrate nitrogen content; NH_4_^+^-N, soil ammonium nitrogen content; AP, soil available phosphorus; MAP, annual mean precipitation; MAT, annual mean temperature; SB-Richness, bacterial richness detected in rhizospheric soil; RB-Richness, bacterial richness detected in tree root; SF-Richness, fungal richness detected in rhizospheric soil; RF-Richness, fungal richness detected in tree root; SB-Composition, bacterial community composition detected in rhizospheric soil using the first axis of NMDS ordination (NMDS1) as a proxy; RB-Composition, bacterial community composition detected in tree root using the first axis of NMDS ordination (NMDS1) as a proxy; SF-Composition, fungal community composition detected in rhizospheric soil using the first axis of NMDS ordination (NMDS1) as a proxy; RF-Composition, fungal community composition detected in tree root using the first axis of NMDS ordination (NMDS1) as a proxy.

# Figure S1


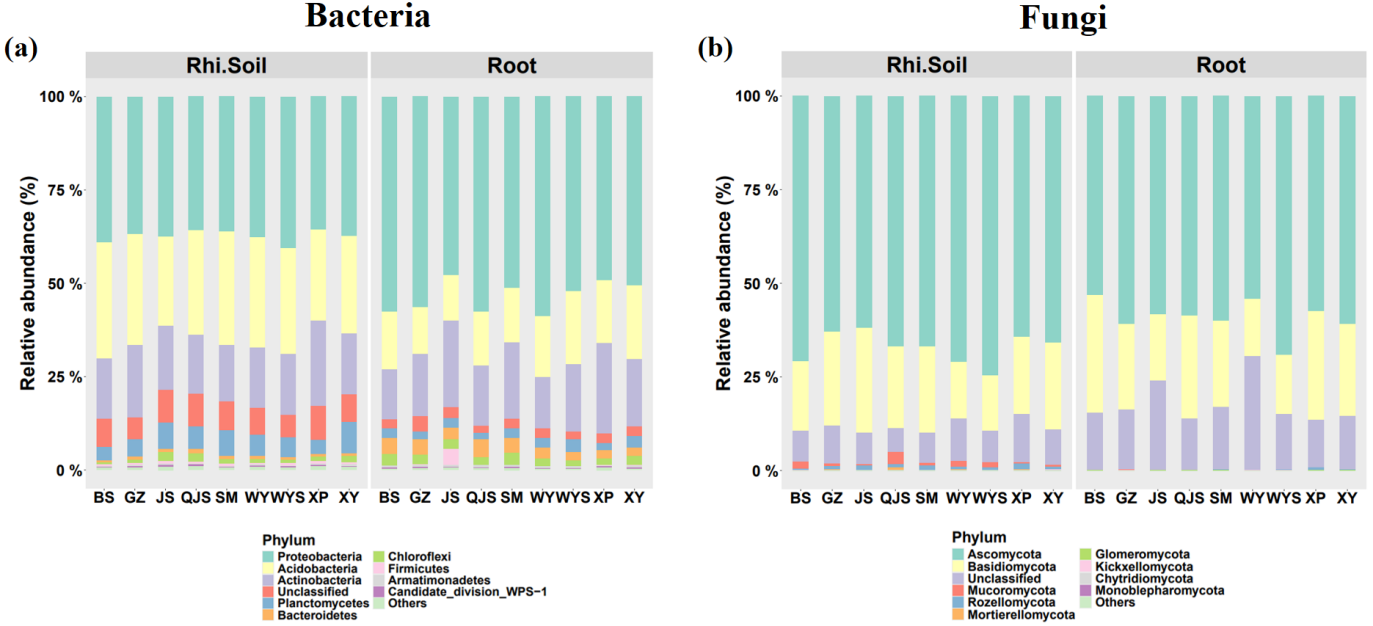


**Fig. S1.** Relative abundance of bacterial (**a**) and fungal (**b**) taxa at the phylum level in rhizospheric soil and root compartments across nine Chinese fir stands. Taxa in the “Others” are the relative abundance of the bacterial and fungal phyla less than top 10 phyla. Abbreviations: BS, Baisha State-owned Forest Farm in Shanghang County; GZ, Guanzhuang State-owned Forest Farm in Sha County; JS, Jinshan State-owned Forest Farm in Hua’an County; QJS, Qiujiashan State-owned Forest Farm in Liancheng County; SM, Fujian Sanming Forest Ecosystem National Observation and Research Station in Sanming City; WY, Wuyi State-owned Forest Farm in Zhangping City; WYS, Wuyishan National Park in Wuyishan City; XP, Xiapu State-owned Forest Farm in Ningde City; XY, Xiayang State-owned Forest Farm in Nanping City.

# Figure S2


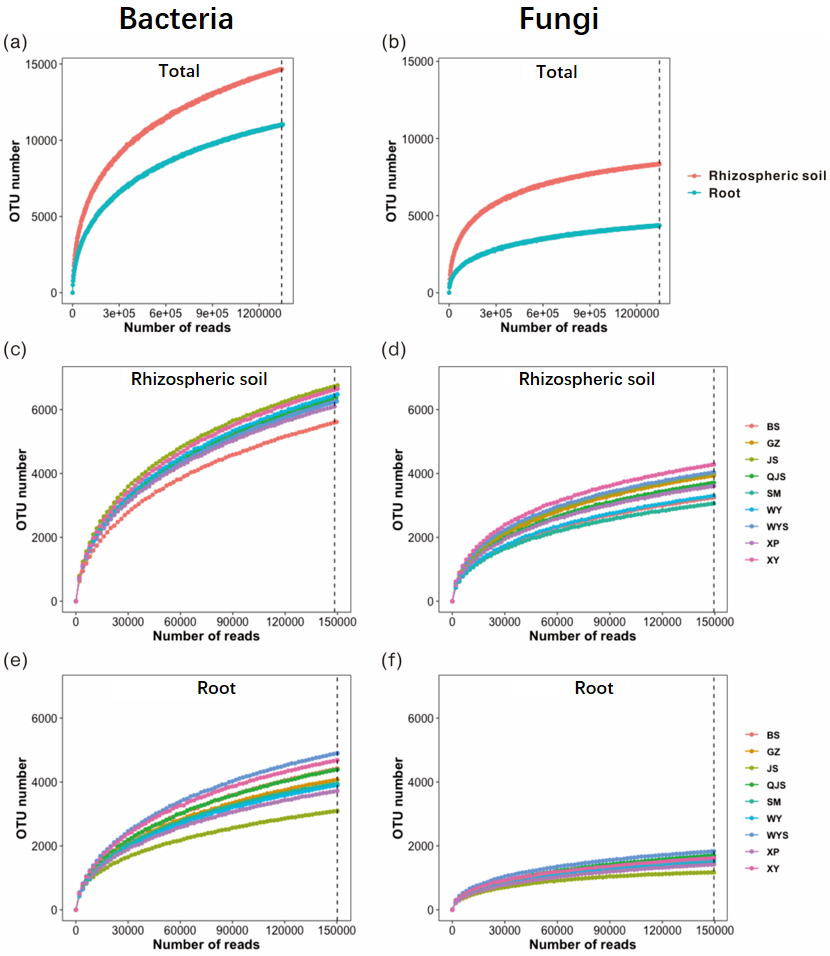


**Fig. S2.** Rarefaction curves for the observed bacterial (**a**) and fungal (**b**) OTUs among two compartments of Chinese fir stands. 108 rhizospheric soil samples of bacteria (**c**) and fungi (**d**) among nine Chinese fir stands, 108 root samples of bacteria (**e**) and 104 root samples of fungi (**f**) among nine Chinese fir stands. Abbreviations: BS, Baisha State-owned Forest Farm in Shanghang County; GZ, Guanzhuang State-owned Forest Farm in Sha County; JS, Jinshan State-owned Forest Farm in Hua’an County; QJS, Qiujiashan State-owned Forest Farm in Liancheng County; SM, Fujian Sanming Forest Ecosystem National Observation and Research Station in Sanming City; WY, Wuyi State-owned Forest Farm in Zhangping City; WYS, Wuyishan National Park in Wuyishan City; XP, Xiapu State-owned Forest Farm in Ningde City; XY, Xiayang State-owned Forest Farm in Nanping City.

# Figure S3


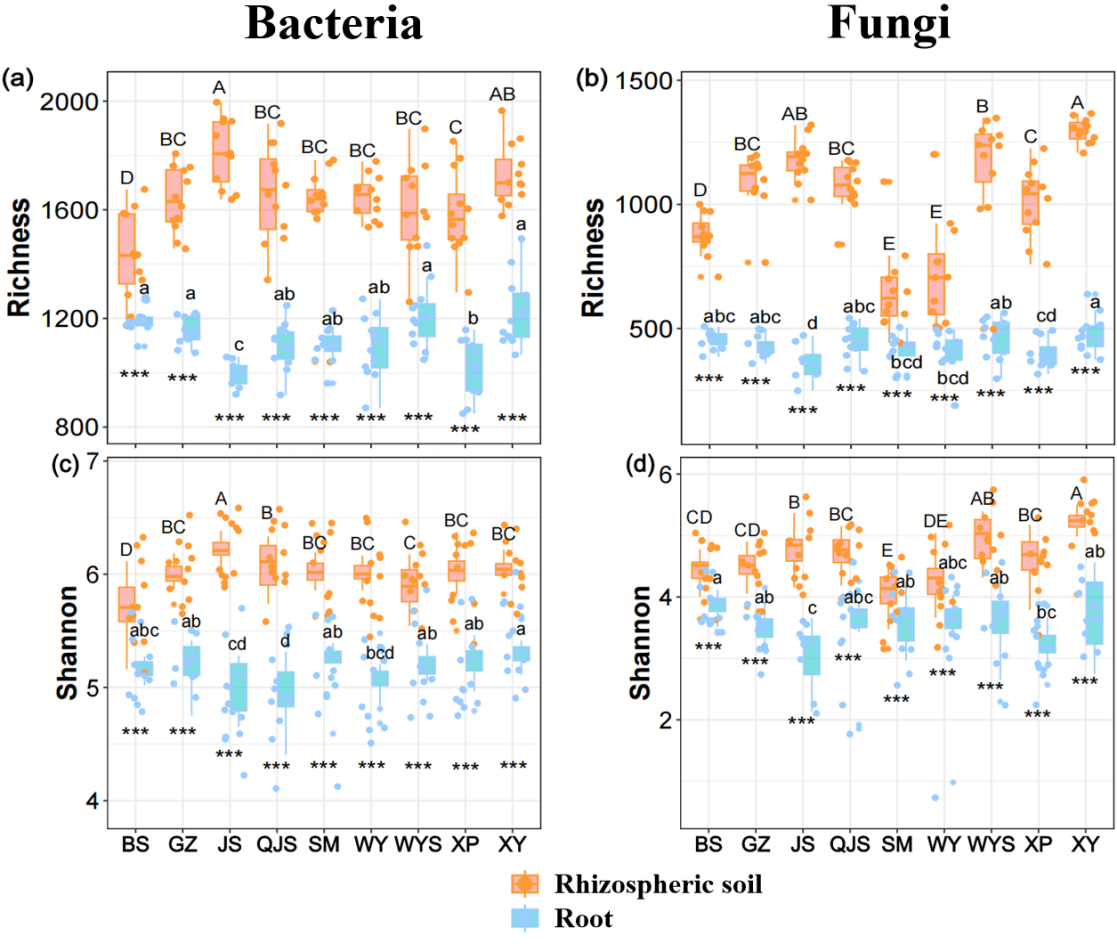


**Fig. S3.** Comparison of rhizospheric soil- and root-associated microbial OTU richness (**a**, bacteria; **b**, fungi) and Shannon index (**c**, bacteria; **d**, fungi) across nine Chinese fir stands. Boxplot centre line indicates the mean, lower and upper hinges the standard deviation around the mean and each whisker correspond to the minimum and maximum values, respectively. Different uppercase and lowercase letters indicate significant differences in OTU richness and Shannon index for rhizospheric soil- and root-associated samples, respectively, across nine plantation locations (P < 0.05). Asterisks indicate levels of significance (**P* <0.05; ***P* < 0.01; ****P* < 0.001). Abbreviations: BS, Baisha State-owned Forest Farm in Shanghang County; GZ, Guanzhuang State-owned Forest Farm in Sha County; JS, Jinshan State-owned Forest Farm in Hua’an County; QJS, Qiujiashan State-owned Forest Farm in Liancheng County; SM, Fujian Sanming Forest Ecosystem National Observation and Research Station in Sanming City; WY, Wuyi State-owned Forest Farm in Zhangping City; WYS, Wuyishan National Park in Wuyishan City; XP, Xiapu State-owned Forest Farm in Ningde City; XY, Xiayang State-owned Forest Farm in Nanping City.

# Figure S4


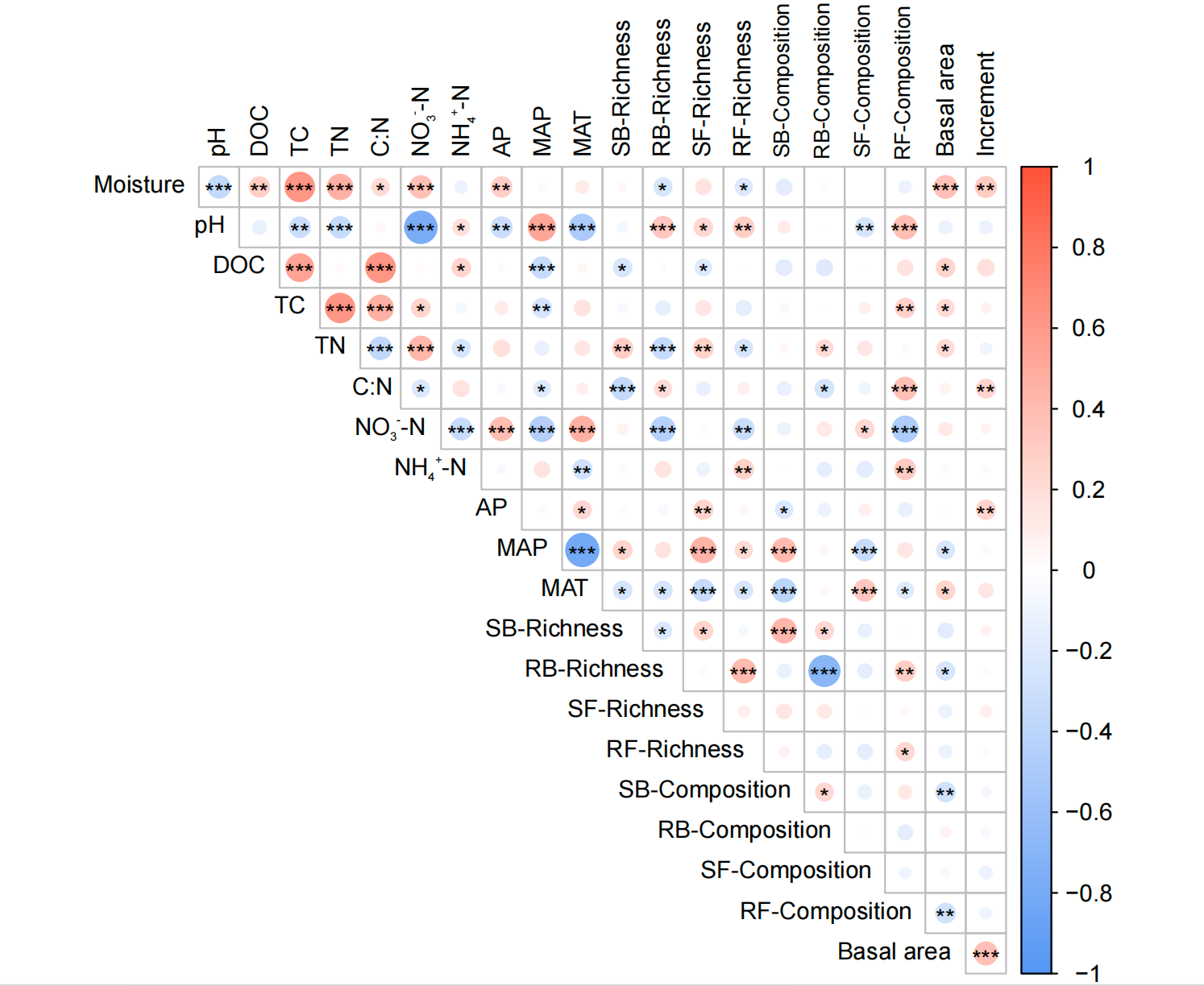


**Fig. S4.** Correlation heat map showing the correlations among the biotic and abiotic factors. Pairwise comparisons of factors are displayed, with a color gradient denoting Pearson’s correlation coefficient. Abbreviations: DOC, soil dissolved organic carbon; TC, soil total carbon; TN, soil total nitrogen; NO_3_^–^-N, soil nitrate nitrogen content; NH_4_^+^-N, soil ammonium nitrogen content; AP, soil available phosphorus; MAP, annual mean precipitation; MAT, annual mean temperature; SB-Richness, bacterial richness detected in rhizospheric soil; RB-Richness, bacterial richness detected in tree root; SF-Richness, fungal richness detected in rhizospheric soil; RF-Richness, fungal richness detected in tree root; SB-Composition, bacterial community composition detected in rhizospheric soil using the first axis of NMDS ordination (NMDS1) as a proxy; RB-Composition, bacterial community composition detected in tree root using the first axis of NMDS ordination (NMDS1) as a proxy; SF-Composition, fungal community composition detected in rhizospheric soil using the first axis of NMDS ordination (NMDS1) as a proxy; RF-Composition, fungal community composition detected in tree root using the first axis of NMDS ordination (NMDS1) as a proxy.
